# Spectrum of bacterial keratitis at a tertiary eye center in Indonesia

**DOI:** 10.1128/spectrum.00234-24

**Published:** 2024-12-10

**Authors:** Rifna Lutfiamida, Rien Widyasari, Kara Citra Kalandra

**Affiliations:** 1 Ocular Infection and Immunology Division, Klinik Mata Nusantara EyeCare, Jakarta, Indonesia; 2 Department of Health Care Management, Ministry of Health Republic of Indonesia, Jakarta, Indonesia; University of Cincinnati, Cincinnati, Ohio, USA

**Keywords:** antibiotic susceptibility, bacterial culture, bacterial keratitis

## Abstract

The purpose of this study is to analyze the bacterial spectrum and antibiotic susceptibility patterns in bacterial keratitis at a tertiary eye center in Jakarta, Indonesia. The study design is a retrospective analytic study. A retrospective cross-sectional analytic review of medical records was conducted for patients with bacterial keratitis who underwent corneal cultures over an 8-year period. The study exclusively included eyes with culture-positive bacterial keratitis. The data were divided into two distinct periods: January 2012 to December 2017 and January 2018 to June 2020. A total of 129 eyes with keratitis were examined, and culture-positive bacteria were found in 74 eyes. Gram-negative bacteria were identified in 65 eyes (69.1%), and Gram-positive bacteria were identified in 29 eyes (30.9%). The most common bacteria identified was *Pseudomonas aeruginosa* (34 positive cultures, 36.2%). Gram-negative bacteria had the highest susceptibility toward ceftazidime (94.7%; 95% CI, 88.9–100). Gram-positive bacteria had the highest susceptibility toward vancomycin (100%; 95% CI, 98.8–100). Gentamicin demonstrated broad sensitivity across all bacterial types (85.7%; 95% CI, 78.2–93.2%), surpassing fluoroquinolones. There were no significant changes in antibiotic susceptibility patterns between the two periods studied (*P* > 0.05). The stability observed in both the bacterial spectrum and antibiotic susceptibility patterns throughout the study period indicates that the current empirical treatment is still effective. However, the results highlight gentamicin as a highly valuable option for the empirical treatment of bacterial keratitis, while targeted treatment with ceftazidime should be considered for Gram-negative infections and vancomycin for Gram-positive infections when culture results are available.

**IMPORTANCE:**

Bacterial keratitis poses a significant public health concern in Indonesia due to its potential for vision loss and morbidity. Understanding the spectrum of bacterial pathogens causing keratitis is crucial for guiding appropriate treatment strategies and improving patient outcomes. This study aims to investigate the prevalence, antibiotic susceptibility patterns, and risk factors associated with bacterial keratitis cases, providing valuable insights for clinicians to optimize management protocols and enhance the overall ocular health landscape in Indonesia or in other countries that have similar geographical conditions.

## INTRODUCTION

Infectious keratitis is a potentially sight-threatening ocular infection caused by various microorganisms, including bacteria, viruses, fungi, and parasites. It represents a major public health challenge, as it is estimated to contribute to 1.5 to 2.0 million new cases of monocular blindness each year (1). This condition is the leading cause of corneal blindness internationally (2–4), and prompt treatment is of paramount importance to preserve corneal integrity and prevent complications.

Bacterial keratitis is the most common type of infectious keratitis in many regions worldwide (2–4). Although several studies have indicated that fungal keratitis is more prevalent in parts of Asia, particularly India (5, 6) and China (7, 8), a multicenter study by the Asia Cornea Society found that bacterial keratitis remains the most common cause of infectious keratitis, diagnosed in 2,521 eyes (38.0%) compared to fungal keratitis in 2,166 eyes (32.7%). *Pseudomonas aeruginosa* was identified as the second most common pathogen (10.7%) following *Fusarium* spp. (18.3%), but it was the most frequently isolated bacterial species in the participating centers across the Philippines, Taiwan, Thailand, and Singapore (9).

There is limited research on the spectrum of bacterial pathogens responsible for keratitis and their antibiotic susceptibility patterns. While broad-spectrum antibiotics such as fluoroquinolones remain the mainstay of empirical therapy, the emergence of antibiotic resistance significantly complicates the management of bacterial keratitis, with susceptibility patterns varying across regions (10). This presents a substantial challenge for clinicians who must often rely on empirical treatment, particularly in areas with limited laboratory resources.

The findings from this study will provide valuable insights into the epidemiology of bacterial keratitis in Indonesia, highlighting the predominant bacterial pathogens and their resistance patterns. This information will help improve empirical treatment strategies in regions with limited laboratory capabilities, ultimately enhancing patient outcomes.

## RESULTS

During the 8-year study period, 129 eyes with keratitis were analyzed, all presenting unilateral involvement. Bacteria were isolated from 74 eyes (57.4%) and fungi from 4 eyes (3.1%), and 55 eyes had negative cultures. The mean patient age was 44.9 ± 22.5 years (range 0–89 years), with the highest incidence in the prime working age group of 24–54 years (47.3%). The study population included 30 males (40.5%) and 44 females (59.5%). Contact lens wear was the most common predisposing factor, affecting 52.3% of cases (Table 1).

From the 74 culture-positive eyes, a total of 94 bacterial strains were identified, with 14 eyes exhibiting polymicrobial infections. These polymicrobial infections involve at least 2 significant bacterial species contributing to the development of bacterial keratitis and were most commonly associated with contact lens wear, found in 10 cases, followed by trauma in 3 cases and blepharitis in 1 case (Table 1).

**TABLE 1:** Demography and predisposing factors

	Total	2012–2017	2018–2020
	*n* (%)		
Number of eyes (%)	74	34 (45.9)	40 (54.1)
Age (years)	44.9 ± 22.5	43.5 ± 20.9	46.0 ± 23.9
Sex (male : female)	30:44	10:24	20:20
Predisposing factors (%)			
Diabetes mellitus	11 (16.9)	4 (12.9)	7 (20.6)
Contact lens wear	34 (52.3)	17 (54.8)	17 (50)
Blepharitis	8 (12.3)	6 (19.4)	2 (5.9)
Ocular trauma history	10 (15.4)	2 (6.5)	8 (23.5)
Ocular surface disease	2 (3.1)	2 (6.5)	0 (0)

Gram-positive bacteria were identified in 29 eyes (30.9%), representing 25.0% of all positive cultures in the first period and 35.2% in the second period. Gram-negative bacteria were identified in 65 eyes (69.1%), accounting for 75.0% of positive cultures in the first period and 64.8% in the second period. Statistical analysis revealed no significant difference in the distribution of Gram-positive and Gram-negative bacteria between the two periods (*P* > 0.05) (Table 2).

**TABLE 2:** Proportion of bacteria isolates

Bacteria	2012–2020 (*n* = 94)	2012–2017 (*n* = 40)	2018–2020 (*n* = 54)	*P* value (Fisher’s exact test)
*Gram positive*	29 (30.9)	10 (25.0)	19 (35.2)	0.11
*Gram negative*	65 (69.1)	30 (75.0)	35 (84.8)	0.923

Gram positive bacteria highest sensitivity was towards vancomycin with 28 out of 28 tests(100%; 95% confidence interval [CI], 98.8-100), followed by gentamicin (83.3% CI 95%,64.5-94.5). Gram negative bacteria highest sensitivity was towards ceftazidime with 54sensitive results out of 57 tests (94.7%; 95% CI, 88.9-100). Similar sensitivity results werealso found in levofloxacin (88.9%; 95% CI, 74.4-100) and gentamicin (86.7%; 95% CI, 78.1-95.3) (Table 3).

**TABLE 3:** Antibiotic sensitivity of all bacterial species, Gram positive and Gram negative isolated from bacterial keratitis^*a*^

Antibiotics		All bacteria			Gram positive			Gram negative	
	n	S	R	n	S	R	n	S	R
Gentamicin	84	85.7 (78.2–93.2)	10.7 (4.1–17.3)	24	83.3 (64.5–94.5)	12.5 (0–25.7)	60	86.7 (78.1–95.3)	10.0 (2.4–17.6)
Ciprofloxacin	76	73.3 (62.9–82.6)	17.1 (9.9–26.8)	23	52.5 (31.8–72.6)	43.5 (23.2–63.7)	53	83.0 (72.9–93.1)	5.7 (0–11.9)
Ceftazidime	60	93.3 (84.7–97.8)	5 (1.3–13)	3	66.7 (13.3–100)	33.3 (0–86.7)	57	94.7 (88.9–100)	3.5 (0.0–8.3)
Cefazolin	44	6.8 (1.7–17.4)	93.2 (82.6–98.3)	1	–	100	43	7.0 (0–14.6)	93 (85.4–100)
Levofloxacin	42	66.7 (51.5–79.6)	28.6 (16.5–43.5)	24	50.0 (30–70)	45.8 (25.9–65.8)	18	88.9 (74.4–100)	5.6 (0–16.1)
Tetracycline	28	53.6 (35.2–71.2)	42.9 (25.7–61.4)	23	65.2 (45.8–84.7)	34.8 (15.3–54.2)	5	–	80 (33.4–99)
Vancomycin	28	100 (98.8–100)	–^*b*^	28	100 (98.8–100)	–	0	–	–

^*a*^ n, sensitive + intermediate + resistant; S, sensitive in % (95% CI); R, resistant in % (95% CI). Not all isolates were tested for all the antibiotics mentioned.

^*b*^ "–",not available.

The most common bacteria identified in this study were *Pseudomonas aeruginosa* (34 positive cultures, 36.2%), *Serratia marcescens* (13 positive cultures, 13.8%), *Staphylococcus aureus* (9 positive cultures, 9.6%), and *Staphylococcus epidermidis* (5 positive cultures, 5.3%). Together, these four groups accounted for 64.9% of all isolates. *Pseudomonas aeruginosa* and *Serratia marcescens*—both Gram-negative bacteria—showed similar susceptibility patterns to ceftazidime and gentamicin. The Gram-positive bacteria, *Staphylococcus aureus* and *Staphylococcus epidermidis*, demonstrated high susceptibility to vancomycin and gentamicin (Table 4).

**TABLE 4:** Antibiotic sensitivity of four most common bacteria isolated from bacterial keratitis^*a*^

Antibiotics	*Pseudomonas aeruginosa*		*Serratia marcescens*		*Staphylococcus aureus*		*Staphylococcus epidermidis*	
	S	R	S	R	S	R	S	R
Gentamicin	34/35 (97.1)	1/35 (2.9)	8/9 (88.9)	0/9 (0.0)	8/10 (80.0)	2/9 (20.0)	4/4 (100)	0/4(0)
Ciprofloxacin	28/30 (93.3)	0/30 (0.0)	8/9 (88.9)	0/9 (0.0)	7/9 (77.8)	2/9 (22.2)	2/4 (50.0)	2/4 (50.0)
Ceftazidime	30/31 (96.8)	1/31 (3.2)	11/12 (91.7)	0/12 (0.0)	–^*b*^	–	–	–
Cefazolin	0/11 (0.0)	11/11 (100)	0/7 (0.0)	7/7 (100)	–	–	–	–
Levofloxacin	9/10 (90.0)	0/10 (0.0)	2/2 (100)	0/2 (0.0)	7/9 (77.8)	2/9 (22.2)	2/4 (50.0)	2/4 (50.0)
Tetracycline	0/4 (0.0)	3/4 (75.0)	–	–	7/9 (77.8)	2/9 (22.2)	2/3 (66.7)	1/3 (33.3)
Vancomycin	–	–	–	–	9/9 (100)	0/9 (0.0)	4/4 (100)	0/4 (0)

^*a*^ S, sensitive (%); R, resistant (%); Intermediate not mentioned. Not all isolates were tested for all the antibiotics mentioned.

^*b*^ "–",not available.

Among the drugs tested against Gram-negative organisms, ceftazidime demonstrated the highest susceptibility at 94.7% (95% CI, 88.9–100%), followed by levofloxacin at 88.9% (95% CI, 74.4–100%), and gentamicin at 86.7% (95% CI, 78.1–95.3). For Gram-positive organisms, vancomycin showed the highest susceptibility with 100% sensitivity (95% CI, 98.8–100%), followed by gentamicin at 83.3% (95% CI, 64.5–94.5). Interestingly, although gentamicin is typically used to treat Gram-negative infections, it also demonstrates notable susceptibility against Gram-positive bacteria. Statistical analysis revealed no significant changes in susceptibility patterns between Gram-negative and Gram-positive bacteria toward the tested antibiotics (*P* > 0.05) (Table 5).

**TABLE 5:** Antibiotic sensitivity of all bacterial species, Gram positive and Gram negative isolated from bacterial keratitis^*a*^

Antibiotics	All bacteria			Gram positive			Gram negative		
	n 2012–2020	S 2012–2017	S 2018–2020	n 2012–2020	S 2017	S 2020	n 2012–2020	S 2012–2017	S 2018–2020
Gentamicin	84	28/36	44/48	24	4/6	16/18	60	24/30	28/30
Cefazolin	44	3/19	0/25	1	–^*b*^	0/1	43	3/19	0/24
Ceftazidime	60	26/28	30/32	3	–	2/3	57	26/28	28/29
Tetracycline	28	4/11	11/17	23	4/8	11/15	5	0/3	0/2
Ciprofloxacin	76	22/32	34/44	23	3/6	9/17	53	19/26	25/27
Levofloxacin	42	19/24	9/18	24	5/8	7/16	18	14/16	2/2
Vancomycin	28	10/10	18/18	28	10/10	18/18	–	–	–

^*a*^ n, sensitive + intermediate + resistant; S, sensitive. Not all isolates were tested for all the antibiotics mentioned.

^*b*^ "–",not available.

## DISCUSSION

The incidence and prevalence of bacterial keratitis are significantly influenced by geographical location, as well as local environmental and climatic conditions. These factors contribute to variations in the types of bacteria that commonly cause infections and the associated patterns of antibiotic resistance. The emergence of antibiotic resistance presents a substantial challenge in managing bacterial keratitis, as resistance patterns can vary widely from one region to another (10). This variability necessitates ongoing surveillance and regional studies to accurately assess local susceptibility patterns and adapt treatment strategies accordingly.

In Indonesia, however, there is limited research on the spectrum of bacterial pathogens responsible for keratitis and their antibiotic susceptibility patterns. This study addresses this gap by providing an overview of the bacterial spectrum and antibiotic susceptibility patterns in keratitis patients within our country. By offering updated insights, this research contributes to a better understanding of local bacterial dynamics and resistance trends, which is essential for optimizing treatment strategies and managing bacterial keratitis effectively.

In this study, culture-positive rates were found in 57.4% of patients with bacterial keratitis, aligning with findings from Lichtinger et al. (57.4%) (11) and Lin et al. (58%) (12). Higher culture-positive rates were reported in studies by Mun et al. (78.3%) (13) and Kaliamurthy et al. (77%) (14). The lower culture-positive rate observed in this study may be attributed to factors such as prior antibiotic treatment and the use of topical anesthetics, both of which can inhibit bacterial growth and potentially affect culture outcomes.

This study also found no significant changes in the prevalence of Gram-positive and Gram-negative bacteria over the study period, which is in line with the observations of Hernandez-Camarena et al. (15) and Oliveira-Ferreira et al. (16). This stability contrasts with the findings of who reported a significant decrease in Gram-positive bacteria and an increase in Gram-negative bacteria, and Marasini et al. (17) reported a significant rise in both bacteria.

*Pseudomonas aeruginosa* emerged as the most prevalent pathogenic bacterium in this study accounting for 36.2% of cases. This finding is consistent with results from studies conducted by Farias et al. (18) (44.62%) from Brazil and Lin et al. (19) (28%) and Hsiao et al. (20) (24.4%) from Taiwan. The prevalence of *Pseudomonas aeruginosa* in our study is closely linked to contact lens wear, which was the most common risk factor (45.9%). This association is also supported by findings from Farias et al. (18) and Lin et al. (19). Previous research in Indonesia has similarly identified *Pseudomonas aeruginosa* as the predominant microorganism in bacterial keratitis, even in the presence of ocular trauma as a predisposing factor. Given these findings and the regional context, *Pseudomonas aeruginosa* may be considered the most common etiological agent of bacterial keratitis in our area (21). The second most common bacterium identified in this study is *Serratia marcescens*, present in 13.8% of cases, and also associated with contact lens wear, consistent with the study by Sadorra et al. (22).

The antibiotic susceptibility patterns for both Gram-negative and Gram-positive bacteria remained stable throughout the study period. Most Gram-negative bacteria exhibited high sensitivity to ceftazidime (94.7%), consistent with findings from Mun et al. (13) and Hsiao et al. (20). In contrast, Hernandez-Camarena et al. (15) reported a significant fivefold increase in resistance of *Pseudomonas aeruginosa* to ceftazidime. They attributed this rise in resistance to the extensive use of ceftazidime as a fortified antibiotic in the empirical treatment of bacterial keratitis.

Despite the high susceptibility toward Gram-positive bacteria to vancomycin (100%) in our study which is consistent with findings from Korea, Taiwan, and the Middle East, where no vancomycin-resistant cases were reported (13, 20, 23, 24), the routine use of vancomycin for the empirical treatment of bacterial keratitis is not common in Indonesia. This preference is influenced by several factors, including the limited availability of vancomycin, the necessity for broad-spectrum antibiotic coverage, and the higher prevalence of Gram-negative bacterial infections.

As a result, broad-spectrum antibiotics like fluoroquinolones remain the preferred empirical therapy in Indonesia due to their availability and effectiveness against a wide range of pathogens. In this study, susceptibility patterns for frequently used fluoroquinolones such as ciprofloxacin and levofloxacin were found to be generally favorable. Although we noted a slight decrease in levofloxacin susceptibility, this change was not statistically significant (*P* > 0.05), similar to a study by Mun et al. (13), and does not diminish its clinical effectiveness.

Aside from fluoroquinolones, gentamicin remains a widely used antibiotic in Indonesia, particularly in rural areas, due to its affordability and accessibility. Our study demonstrated that gentamicin exhibits high sensitivity to both Gram-negative (86.7% 95% CI, 78.1–95.3) and Gram-positive (83.3% 95% CI, 64.5–94.5) bacteria, surpassing fluoroquinolones in effectiveness across all bacterial types (85.7% 95% CI, 78.2–93.2). It showed strong efficacy against *Pseudomonas aeruginosa* (97.1%) and *Serratia marcescens* (88.9%), which are among the most common pathogens identified in our study. Although traditionally used primarily for Gram-negative infections, gentamicin’s significant activity against Gram-positive bacteria underscores its versatility. This finding is consistent with other research, including studies by Marasini et al. (17) and Ting et al. (25), which reported that gentamicin is effective against a broad spectrum of bacterial pathogens, with efficacy exceeding 95%.

In conclusion, this study indicates that Gram-negative bacteria are the predominant pathogens associated with bacterial keratitis. The stability observed in both the bacterial spectrum and antibiotic susceptibility patterns throughout the study period indicates that current empirical treatment of fluoroquinolones is still effective although continuous surveillance is essential to detect any emerging shifts that could impact treatment efficacy.

Our study’s limitations include its retrospective design and relatively small sample size, which suggest the need for further research to confirm these findings. Nonetheless, the results highlight gentamicin as a highly valuable option for the empirical treatment of bacterial keratitis, especially in rural areas where its affordability and broad-spectrum efficacy provide significant benefits. While gentamicin is a practical choice for empirical therapy, targeted treatment with ceftazidime should be considered for Gram-negative infections and vancomycin for Gram-positive infections when culture results are available. This tailored approach ensures optimal management of bacterial keratitis based on specific bacterial profiles and resistance patterns.

## MATERIALS AND METHODS

Data acquisition and study approval were granted by the ethics committee and research board of our institution. A retrospective cross-sectional analytic review of medical records was performed for patients with bacterial keratitis who visited our tertiary eye center and underwent corneal cultures over an 8-year period.

The study exclusively includes patients with comprehensive medical records who tested positive for bacterial cultures. The data is divided into two periods: January 2012 to December 2017 and January 2018 to June 2020. This division was based on our tertiary eye center’s standard operating procedures for managing bacterial keratitis cases, which was implemented from January 2018 onward.

Before January 2018, bacterial culture testing was conducted only on patients who did not improve with empirical fluoroquinolone therapy within 48 hours, representing a purposive sampling approach. In contrast, from January 2018 onward, the standard operating procedures were revised to include bacterial culture testing for all patients diagnosed with bacterial keratitis, thereby shifting to a more inclusive sampling method.

Bacterial culture specimens were collected using Amies transport medium under direct visualization with a slit‐lamp biomicroscope. Bacterial inoculation was then performed in a laboratory at 37.8°C on defibrinated 5% sheep blood agar and chocolate agar or fluid thioglycollate medium. Cultures were evaluated by an experienced laboratory staff member at 24 and 48 hours, and a positive result was determined as the growth of microorganisms along the line of inoculation on at least one medium and subsequently discarded if there was no growth. The bacterial isolates were then tested for identification and antimicrobial susceptibility with an automated Vitek-2 system, and results were then validated by microbiologists according to the CLSI M100 breakpoints in which year the specimens were taken and categorized as sensitive, intermediate, or resistant toward the tested antibiotics. Antibiotic agents evaluated were gentamicin, cefazolin, ceftazidime, tetracycline, ciprofloxacin, levofloxacin, and vancomycin.

The analysis revealed no significant differences in the CLSI M100 breakpoints for the bacterial isolates and antibiotic agents evaluated throughout the study. The only exceptions were the disk diffusion and minimal inhibitory concentration breakpoints for ciprofloxacin and levofloxacin, as outlined in the CLSI M100 29th Edition. These breakpoints have been effectively applied in the laboratory by microbiologists.

Chi-square test and the alternative Fisher’s exact test were used for the statistical analysis and calculated with SPSS (version 20.0; IBM Corp., New York, NY, USA). *P* value of <0.05 was considered statistically significant.
